# A life-threatening arrhythmia detection method based on pulse rate variability analysis and decision tree

**DOI:** 10.3389/fphys.2022.1008111

**Published:** 2022-10-14

**Authors:** Lijuan Chou, Jicheng Liu, Shengrong Gong, Yongxin Chou

**Affiliations:** ^1^ School of Electrical and Automatic Engineering, Changshu Institute of Technology, Suzhou, China; ^2^ School of Computer and Information Technology, Northeast Petroleum University, Daqing, China; ^3^ School of Computer Science and Engineering, Changshu Institute of Technology, Suzhou, China

**Keywords:** pulse rate variability, arterial blood pressure, cardiovascular diseases, life-threatening arrhythmias, decision tree, intelligent recognition

## Abstract

Extreme bradycardia (EB), extreme tachycardia (ET), ventricular tachycardia (VT), and ventricular flutter (VF) are the four types of life-threatening arrhythmias, which are symptoms of cardiovascular diseases. Therefore, in this study, a method of life-threatening arrhythmia recognition is proposed based on pulse rate variability (PRV). First, noise and interference are wiped out from the arterial blood pressure (ABP), and the PRV signal is extracted. Then, 19 features are extracted from the PRV signal, and 15 features with highly important and significant variation were selected by random forest (RF). Finally, the back-propagation neural network (BPNN), extreme learning machine (ELM), and decision tree (DT) are used to build, train, and test classifiers to detect life-threatening arrhythmias. The experimental data are obtained from the MIMIC/Fantasia and the 2015 Physiology Net/CinC Challenge databases. The experimental results show that the DT classifier has the best average performance with accuracy and kappa coefficient (*kappa*) of 98.76 ± 0.08% and 97.59 ± 0.15%, which are higher than those of the BPNN (*accuracy* = 94.85 ± 1.33% and *kappa* = 89.95 ± 2.62%) and ELM (*accuracy* = 95.05 ± 0.14% and *kappa* = 90.28 ± 0.28%) classifiers. The proposed method shows better performance in identifying four life-threatening arrhythmias compared to existing methods and has potential to be used for home monitoring of patients with life-threatening arrhythmias.

## 1 Introduction

In recent years, cardiovascular diseases have the highest mortality rate and are the “number one killer” of human beings (Roberts and Fair 2021). Among them, acute cardiovascular diseases such as myocardial infarction (MI) and cerebral infarction (CI) have the high suddenness and lethality (Du et al., 2021). If MI and CI are not effectively treated within a few hours after the sudden onset, it will directly lead to the patient’s death.

Life-threatening arrhythmias are a common symptom in patients with CI and MI, and the common life-threatening arrhythmias include EB, ET, VT, and VF (Deaconu et al., 2021), and the definitions of those four life-threatening arrhythmias are given in Table 1 according to the beating rhythm of the heart rate (Alinejad et al., 2019; Paliakaitė et al., 2019). In the initial period of suddenness of life-threatening arrhythmias, patients sometimes experience sudden heart pain that is slight and rapid and disappears after a short rest such as sitting or lying down, which is called “transient” (Chorin et al., 2021). The “transient” of life-threatening arrhythmias is often ignored by patients, which can lead to the sudden illness of dangerous MI, CI, and other acute cardiovascular diseases. On the eve of acute cardiovascular diseases such as MI and CI, significant abnormal changes in physiological parameters such as electrocardiographic (ECG) and blood pressure occur (Jahmunah et al., 2021; Shuvo et al., 2021). Moreover, if these abnormalities can be monitored in time, then patients can be warned of the risk so that they can seek medical help, which would significantly reduce the rate of death from acute cardiovascular disease.

**TABLE 1:** Types and definition of four life-threatening arrhythmias.

Types	Definition
EB	HRV <40 bpm for 5 consecutive beats
ET	HRV >140 bpm for 17 beats
VT	Five or more ventricular beats with HRV >100 bpm
VF	Rapid flutter, oscillatory, or fibrillation lasting at least 4 s

At present, the main detection method is hospital ECG, while the acquisition of ECG signal requires multiple electrodes and cable connection and the process needs professional medical staff’s guidance. If one electrode is wrongly attached, the whole signal is no longer valuable. In addition, the prolonged electrode connection can cause skin irritation (Chou et al., 2019). It is difficult for short-time ECG monitoring to effectively recognize life-threatening arrhythmias with transient; thus, long-term tracking and detection of physiological signals is required to achieve recognition of acute cardiovascular disease outbreaks.

The beat rhythm of the heart is transmitted to the pulse with the blood, and both ECG and pulse period sequences can effectively reflect heartbeat rhythm changes (Mitchell and Schwarzwald, 2021). Heart rate variability (HRV) is calculated from ECG, which reflects the rate of the heartbeat and is used to assess the autonomic nervous system of the heart (Ishaque et al., 2021); thus, HRV can be used for the diagnosis of cardiovascular diseases (Saul and Valenza, 2021). The PRV is extracted from the ABP signal, which reflects the subtle changes in the vascular pulse cycle (Jan et al., 2019). Moreover, the PRV can be utilized to assess cardiovascular autonomic activity (Mejía-Mejía et al., 2021). Studies have shown that the PRV extracted from the ABP signal and HRV obtained from the ECG signal have a strong correlation and are interchangeable in cardiovascular disease monitoring in the supine or resting state (Mejía-Mejía et al., 2020; Hejjel and Béres, 2021). Compared with the ECG signal, the ABP signal acquisition does not require the affixing of multiple electrodes and can be easily affixed to multiple parts of the body, which is easy to operate and can be self-measured (Jan et al., 2019; Mejía-Mejía et al., 2022). Thus, ABP signals are widely used in wearable devices such as bracelets and smart watches (Zhu et al., 2021). The study of the life-threatening arrhythmia detection method based on the PRV signal is expected to be used for home monitoring of life-threatening arrhythmias.

Therefore, based on the PRV signal, this study studies techniques for the recognition of four life-threatening arrhythmias: EB, ET, VT, and VF. First, the interference and noise in the pulse signal are filtered out, and then, the PRV signal is extracted from ABP. Next, the parameters of physiological and pathological changes caused by these four life-threatening arrhythmias are extracted from the PRV signal, and the parameters with high importance and contribution are obtained as feature vectors by RF to train classifiers of BPNN, ELM, and DT to detect these four life-threatening arrhythmias.

This study is structured as follows: Section 2 gives the experimental data we used and describes the process and methods of the experiments; Section 3 describes the experimental results, including signal preprocessing, PRV extraction, feature parameter extraction and dimensionality reduction, and classification results; the discussion of the experimental results is given in Section 4; and the conclusions of the study are presented in Section 5.

## 2 Materials and methods

### 2.1 Materials

The experimental data consisted of two groups, both of them from the international physiological signal database: *PhysioBank*. One group has 10 young (aged 21–31) and 10 elderly (aged 70–85) healthy subjects with equal males and females, which comes from the sub-database “MIMIC/Fantasia” (Iyengar et al., 1996; Goldberger et al., 2000). The other group has patients with four life-threatening arrhythmias consisting of 17 EB, 39 ET, 47 VT and 6 VF subjects, which comes from the sub-database “2015 Physiology Net/CinC Challenge” (Clifford et al., 2015).

The data of healthy subjects: the data of healthy subjects are the PRV signal extracted from the ABP signal in the MIMIC/Fantasia database. Before the data recording, every non-smoking subject underwent a physical examination, and only the healthy subjects were allowed to participate. In addition, each recording includes the continuous ECG, respiration, ABP signals with a sampling rate of 250 Hz, and a duration of 2 h.

The data of patients with four life-threatening arrhythmias: the data are the PRV signal extracted from the ABP signal in the 2015 Physiology Net/CinC Challenge database, which was recorded from patients in the intensive care unit of hospitals. During data recording, two ECGs and one ABP signal were collected from the patients, and all signals were sampled at 250 Hz with a duration of 5 or 5.5 min.

The simulation software is MATLAB 2020b, installed on an Intel(R) Core (TM) i5-6300HQ CPU at 2.30 GHz, Windows-10 64-bit operating system, and installed on a laptop with 8 GB RAM.

### 2.2 Methods

**FIGURE 1:**
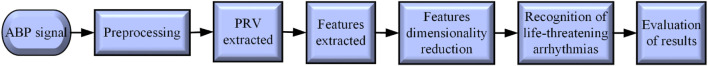
Process of four life-threatening arrhythmias.

Figure 1 depicts the processing of the intelligent recognition of those four life-threatening arrhythmias, which includes six steps: the preprocessing of the ABP signal, extraction of PRV, extraction of features, dimensionality reduction of features by RF, life-threatening arrhythmia recognition, and evaluation of results. The details are displayed in the following subsections.

#### 2.2.1 The preprocessing of the arterial blood pressure signal

**FIGURE 2:**
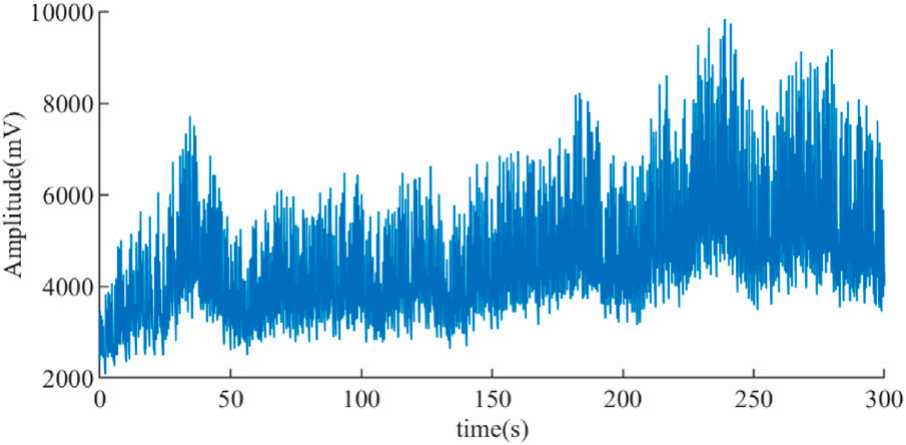
ABP signal of an ET patient with noise.

Noise such as electromyographic (EMG) interference, alternating current (AC) interference, and baseline drift can be generated in the ABP signal acquisition, for example, the ABP signal from an ET patient with noise is displayed in Figure 2. The purpose of ABP signal preprocessing is to wipe out these noises and obtain a clean ABP signal in order to improve the accuracy of PRV extraction. According to the range of frequencies, an integer coefficient notch filter with a stop frequency of 0 Hz, 50 Hz, and its integer multiples are used for de-noising the AC interference and the baseline drift, and an integral coefficient low-pass filter is utilized to eliminate the EMG interference in this study (Chou et al., 2020).

The transfer function *F*_*1*_(*Z*) of the integer coefficient notch filter is,

$$F_{1}\left(Z\right)=F_{AP}\left(Z\right)-F_{BP}\left(Z\right)=Z^{-\left(R-P\right)\cdot \frac{N}{2}}-{\left[\frac{1-Z^{-R}}{Q\left(1-Z^{-P}\right)}\right]}^{N}.$$ (1)

In Equation 1, *F*_*AP*_(*Z*) is the transfer function of the all-pass filter; *F*_*BP*_(*Z*) is the transfer function of the band pass filter; *N* is the order of the filter; R and P are the order of the numerator polynomial and denominator polynomial of the transfer function, respectively, where *P* = *f*_*s*_/*f*_*1*_, *f*_*s*_ is the sampling rate of the signal and is 250Hz and *f*_*1*_ is the notch frequency and is 50 Hz here; and *Q* is the gain of the filter (i.e., the amplification) and should be 2^*N*^, which is proportional to the steepness of the notch band, and *Q* = *R*/*P*. In this study, *N* = 2, *P* = 5, and *Q* = 64 by trial and error, and R = PQ = 320. Therefore, Equation 1 becomes

$$F_{1}\left(Z\right)=Z^{-315}-{\left[\frac{1-Z^{-320}}{64-64Z^{-5}}\right]}^{2}=\frac{-1+4096Z^{-315}-8190Z^{-320}+4096Z^{-325}-Z^{-640}}{4096\left(1-2Z^{-5}+Z^{-10}\right)}.$$ (2)

Then, Equation 3 is the difference equation, which is calculated to eliminate the ABP signal containing the AC interference and the baseline drift in real time by Equation 2.

$$y_{1}\left(n\right)=2y_{1}\left(n-5\right)-y_{1}\left(n-10\right)+\frac{1}{4096}\left[-x_{1}\left(n\right)+4096x_{1}\left(n-315\right)-8190x_{1}\left(n-320\right)+4096x_{1}\left(n-325\right)-x_{1}\left(n-640\right)\right],$$ (3)

where *x*_*1*_(*n*) is the latest data of the ABP signal, *x*_*1*_ (*n-r*) is the *r*-th sampling data before *x*_*1*_(*n*), and *y*_*1*_(*n*) is the output of the integer coefficient notch filter.

The frequency response of the integer coefficient notch filter is

$$H_{1}=e^{-\frac{jwN\left(R-P\right)}{2}}-{\left[\frac{1-\sin\frac{wR}{2}}{Q\sin\frac{wP}{2}}\right]}^{N}=e^{-315jw}-{\left[\frac{1-\sin160w}{64\sin\frac{5w}{2}}\right]}^{N}.$$ (4)

**FIGURE 3:**
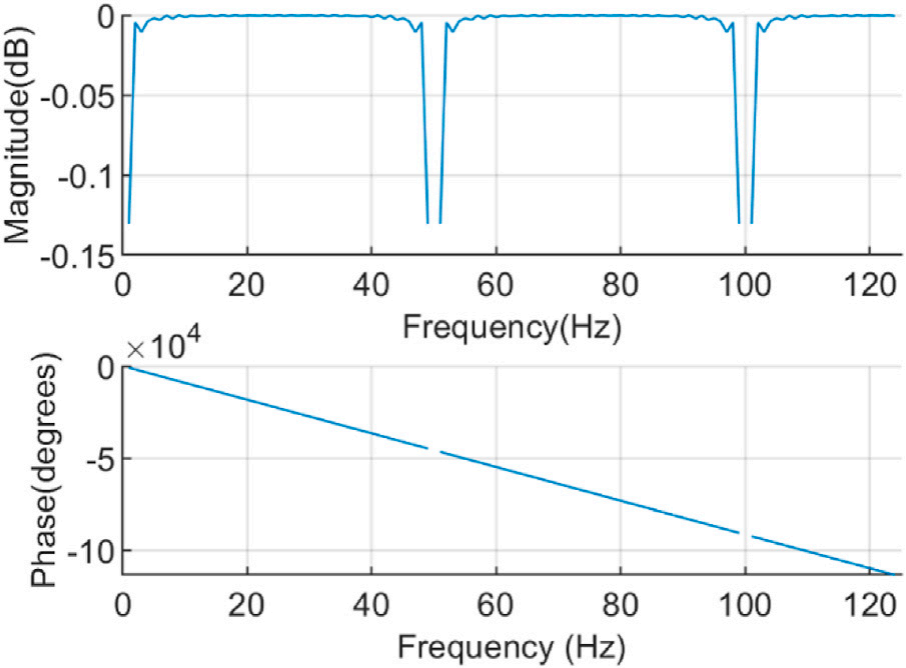
Frequency response of the integer coefficient notch filter.

The frequency response is illustrated in Figure 3. The filter with notch frequencies of 0Hz, 50Hz, and 100 Hz can effectively de-noise the AC interference and the baseline drift, and it has linear phase in the passband.

The transfer function *F*_*2*_(*Z*) of integer coefficient low-pass filter is

$$F_{2}\left(Z\right)={\left[\frac{1-Z^{-C}}{C\left(1-Z^{-1}\right)}\right]}^{N},$$ (5)

where *N* is the order of the filter, *f*_*s*_ is the sampling frequency, *f*_*2*_ is the first-order cut-frequency, and *C* must be an integer and is *f*_*s*_/*f*_*2*_. Here, *f*_*s*_ = 250Hz, *f*_*2*_ = 62.5Hz, *N* = 2, so C = 4. Therefore, Equation 5 becomes

$$F_{2}\left(Z\right)={\left[\frac{1-Z^{-4}}{4\left(1-Z^{-1}\right)}\right]}^{2}.$$ (6)

Then, as displayed in Equation 7, the difference equation is calculated to de-noise the ABP signal containing the EMG interference in real time.

$$y_{2}\left(n\right)=y_{2}\left(n-1\right)+\frac{1}{4}\left[x_{2}\left(n\right)-x_{2}\left(n-4\right)\right],$$ (7)

where *x*_*2*_(*n*) is the latest datum of the ABP signal, *x*_*2*_ (*n-c*) is the *c*-th sampling datum before *x*_*2*_(*n*), and *y*_*2*_(*n*) is the output of the integer coefficient low-pass filter.

The frequency response of the integer coefficient low-pass filter is

$$H_{2}={\left[\frac{1}{C}e^{-\frac{j\left(C-1\right)}{2}}\frac{\sin\left(\frac{wC}{2}\right)}{\sin\left(\frac{w}{2}\right)}\right]}^{N}={\left[\frac{1}{4}e^{-\frac{3j}{2}}\frac{\sin\left(2w\right)}{\sin\left(\frac{w}{2}\right)}\right]}^{2}.$$ (8)

**FIGURE 4:**
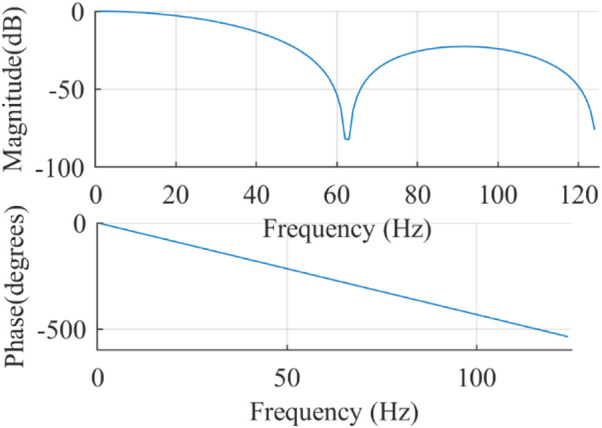
Frequency response of the integer coefficient low-pass filter.

The frequency response is illustrated in Figure 4. The filter with a stop band frequency of 62.5 Hz can effectively suppress the EMG interference, and it has linear phase in the passband.

#### 2.2.2 The extraction of pulse rate variability

**FIGURE 5:**
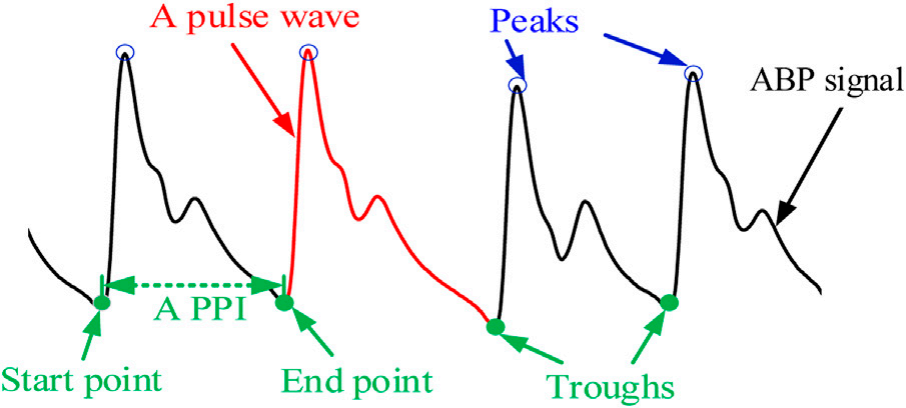
ABP signal.

Since the cardiac cycle corresponds to the pulsation period, one heartbeat produces one pulse wave. An ABP signal consisting of a series of pulse waves (the red curve) is displayed in Figure 5. The start and end points are two pulse troughs (the solid green dots) corresponding to a complete pulse wave, respectively, where the end point of one pulse wave is the start point of the next pulse wave. In general, the PRV can be calculated by computing the first-order difference between the start points and end points, that is, pulse-to-pulse intervals (PPIs). However, it is not easy to detect troughs due to the small amplitude of the waves corresponding to the pulse troughs, while the waves corresponding to the pulse peaks (the blue hollow cycle) have notable characteristics and are easy to detect. Therefore, in this study, the frequency domain extraction method based on sliding window iterative discrete Fourier transform (SWIDFT) is used to detect the wave peaks (Chou et al., 2014), which can be corrected using a manual calibration method if there are incorrect or missing sampling points. Two adjacent pulse peaks are utilized as the boundary to divide the PRV signal, which is calculated by the time interval between two adjacent peaks, and the equation is as follows

$$PRV\left(i\right)=\frac{60}{t}=\frac{60\cdot f_{s}}{Peaks\left(i+1\right)-Peaks\left(i\right)},$$ (9)

where *t* is the sampling time of the ABP signal, and *f*_*s*_ is the sampling frequency of the ABP signal.

#### 2.2.3 Pulse rate variability feature extracted

So far, the main methods for analyzing physiological signals include time domain analysis, frequency domain analysis, and nonlinear domain analysis, from which some features are extracted to describe changes in heartbeat rhythm for the diagnosis of cardiovascular diseases (Sluyter et al., 2019; Mandal et al., 2021). In this study, 19 features were extracted from the PRV signal based on the description of heart rhythm changes.

##### 2.2.3.1 Feature extraction based on time domain analysis

The PRV signal is quantified in the time domain, and some useful information is extracted from the PRV signal by the statistical analysis method to analyze the temporal variation among the PRV signal and obtain the abnormalities and stability of the cardiovascular system. We extracted seven indexes in the time domain, which are calculated as follows.

1) *Mean*: the average of the PRV signal, and the equation is

$$Mean=\overset{n}{\underset{i=1}{\sum }}\frac{S\left(i\right)}{n},$$ (10)

where *S(i)* is the *i*th datum of the PRV signal, and *n* is the length of the PRV signal.

2) *Std*: the standard deviation of the PRV signal, which can reflect the dispersion of the *Mean* and the datum of the PRV signal. The equation is

$$Std=\sqrt{\frac{1}{n-1}\overset{n}{\underset{i=1}{\sum }}{\left(S\left(i\right)-Mean\right)}^{2}.}$$ (11)

3) *RMSD*: the root mean square of PRV signal’s difference, which can reflect the degree of rapid change in the PRV signal. The equation is

$$RMSD=\sqrt{\frac{1}{n-1}\overset{n-1}{\underset{i=1}{\sum }}{\left(S\left(i+1\right)-S\left(i\right)\right)}^{2}.}$$ (12)

4) *nRMSD*: the normalized *RMSD*, and the equation is

$$nRMSD=\frac{RMSD}{Mean}.$$ (13)

5) *PNN40*: the percentage of difference in time intervals between adjacent sampling points of a PRV signal greater than 40 ms. The higher the value, the higher the nervous system tension. The equation is

$$PNN40=\frac{NN40}{TotalNN}\times 100\%,$$ (14)

where *NN40* is the number of time intervals between two adjacent sampling points in a PRV signal that exceed 40 ms, and *TotalNN* is the number of sampling points intervals of a PRV signal.

6) *PNN70*: the percentage of difference in time intervals between adjacent sampling points of a PRV signal greater than 70 ms. The equation is

$$PNN70=\frac{NN70}{TotalNN}\times 100\%,$$ (15)

where *NN70* is the number of time intervals between two adjacent sampling points in a PRV signal that exceed 70 ms.

7) *Mid*: the median of the PRV signal, which represents a value in the PRV signal distribution that can divide the PRV signal into two groups. For a sequence of PRV signal from small to large, when *n* is an odd number, the equation is

Mid=S((n+1)/2). (16)

When *n* is an even number, the equation is

$$Mid=\frac{S\left(n/2\right)+S\left(n/2+1\right)}{2}.$$ (17)

8) *IQR*: the interquartile range of the PRV signal, which describes the dispersion of the PRV signal. The equation is

$$IQR=\frac{S_{75}}{S_{25}},$$ (18)

where S_75_ is the third quartile, and S_25_ is the first quartile.

9) *RMSD_APM*: the root mean square of amplitude’s (APM) difference, which can reflect the degree of rapid change in APM. The calculation method is the same as Equation 12.

##### 2.2.3.2 Feature extraction based on frequency domain analysis

The power spectrum is calculated using the autoregressive (AR) model for the PRV signal, from which the features are extracted according to the frequency range to reflect the stability of cardiovascular activity within the human body and to obtain information about the variability of the cardiovascular system (Fallet et al., 2019).

1) *LF_HF*: the ratio of low frequency (*LF*) to high frequency (*HF*), which can reflect a balanced state of sympathetic and parasympathetic tone.

$$LF_{\_}HF=\frac{LF}{HF}$$, (19)

where the range of *LF* is 0.04–0.15, and the range of *HF* is 0.15–0.4.

##### 2.2.3.3 Feature extraction based on nonlinear domain analysis

The methods of nonlinear domain analysis are Poincaré plot (Nordin et al., 2019) and entropy (Rohila and Sharma, 2019), where the Poincaré plot can be approximated as an ellipse with the horizontal axis of a single time interval of the PRV signal and the vertical axis of time interval of two adjacent PRV signals, which can be utilized to reflect the variation of different PRV signals. The following are some relevant features of the calculation.

1) *Sd*_*1*_ *:Sd*_*2*_: the ratio of the long half-axis (*Sd*_*1*_) to the short half-axis (*Sd*_*2*_) of the ellipse. The equation is

$$S{d_{1}}_{\_}Sd_{1}=\frac{Sd_{1}}{Sd_{2}},$$ (20)

where *Sd*_*1*_ is defined as

$$Sd_{1}=\sqrt{\frac{1}{n-1}\overset{n-1}{\underset{i=1}{\sum }}\frac{{\left(S\left(i+1\right)-S\left(i\right)\right)}^{2}}{2}},$$ (21)

and *Sd*_*2*_ is defined as

$$Sd_{2}=\sqrt{\frac{1}{n-1}\overset{n-1}{\underset{i=1}{\sum }}\frac{{\left(S\left(i+1\right)+S\left(i\right)-2Mean\right)}^{2}}{2}}.$$ (22)

2) *Se*: the area of the ellipse is

$$Se=\prod \cdot Sd_{1}\cdot Sd_{2}.$$ (23)

3) *TPR_PR*: the turning point ratio of the PRV signal, which can measure the randomness of the PRV signal. The equation is

$$TPR_{\_}\mathrm{PR}=\frac{Num\left(\left(S\left(i\right)-S\left(i-1\right)\right)\cdot \left(S\left(i\right)-S\left(i+1\right)\right)>0\right)}{n},$$ (24)

where *Num* is used to count the number of turning point.

4) *ShE*: the Shannon entropy of the PRV signal, and the equation is

$$ShE=-\overset{n}{\underset{i=1}{\sum }}P\left(i\right)\log_{2}\left(P\left(i\right)\right),$$ (25)

where *P(i)* is the probability of the *i*-th datum in the PRV signal.

5) *SamE_PR*: the sample of the PRV signal, and the calculation is as follows:

$$SamE_{\_}\mathrm{PR}=-\ln\frac{B^{m+1}\left(r\right)}{B^{m}\left(r\right)},$$ (26)

where *B* ^*m*^(*r*) is the average probability of the PRV signal when the embedding dimension is *m*, and *B* ^*m+1*^(*r*) is the average probability of the PRV signal when the embedding dimension is *m+1*. Here, *m* is equal to 2.

6) *CSampEn*: the coefficients of sample entropy, and the calculation is as follows (Eerikäinen et al., 2018)

CSampEn=SamE_PP+ln(2r)−ln(mean(S)), (27)

where *r* is the tolerance and is equal to 0.25 here, and *S* is the PRV signal in the buffer.

7) *PE_PR*: the permutation entropy of the PRV signal, and the equation is

$$PE\_\mathrm{PR}=-\overset{m!}{\underset{i=1}{\sum }}P\left(i\right)\log\left(P\left(i\right)\right),$$ (28)

where *P*(*i*) is the probability of occurrence of mode *i*.

8) *SamE_APM:* the sample entropy of APM, and the calculation method is the same as that of Equation 26.
9) *TRP_APM:* the turning point ratio of APM, and the calculation method is the same as that of Equation 24.

#### 2.2.4 Feature dimensionality reduction

In this study, a method of RF is used to measure the importance of the feature parameters and to reduce the feature dimensionality with less information loss (Qi, 2012), which will make the subsequent recognition of life-threatening arrhythmias more efficient without overfitting due to too many features. The method of RF in the “neural network toolbox” of MATLAB 2020b is exploited to calculate the importance of feature parameters for feature dimensionality reduction. The related functions are as follows:

*RF_model* = classRF_train (*p_train, t_train, ntree, mtree, extra_options*);
*Feature_measure* = *RF_model.*importance.

The function “classRF_train” is engaged to train the RF model. The input parameters *p_train* and *t_train* are the features and labels of the training set, respectively. The input parameter *ntree* is the number of trees; here, it is 100. The input parameter *mtree* is the number of predictors used for segmentation at each node; here, it is a rounding down for the number of features, that is, 4. The input parameter *extra_options* is used to control the RF model.

The function “RF_model.importance” allows to calculate feature weights using accuracy and Gini index. The accuracy and Gini index reflect the importance of the features, and the larger the value of accuracy and Gini index, the more important the feature is.

#### 2.2.5 Life-threatening arrhythmia classification

In this study, supervised learning methods, which include BPNN, ELM, and DT, are engaged to design classifiers to identify four life-threatening malignant arrhythmias. The BPNN and DT classifiers are built, trained, and tested with the “neural network toolbox” in MATLAB 2020b. In addition, the classification performance is analyzed using Kappa coefficients, accuracy, and time consumption.

##### 2.2.5.1 BPNN classifier

A BPNN classifier consists of an input layer, one or more hidden layers, and an output layer. After entering the training set into the input layer, the training set is calculated by weights and thresholds in the hidden layer, and the result is transported to the output layer to calculate a prediction value. If the error between the predicted value and the expected value is too large, the error is passed to the input layer and calculated again until the predicted value and the expected value meet the requirements (Hamdani et al., 2022). The BPNN classifier is composed of the following three functions:

*Net* = feedforwardnet (*option*);
*Net_BP* = train (*Net, p_train, t_train*);
*Error_sim_BP* = sim (*Net_BP, p_test*).

The function “feedforwardnet ( )” is utilized to build the BPNN classifier. The *option* is the number of nodes in every layer of the BPNN; here, the number of nodes in one input layer, two hidden layers, and one output layer is 5, 15, 15, and 1, respectively. In addition, the number of training sessions, the minimum error of the training target, and the learning rate are set to 3,000, 0.001, and 0.1, respectively. The training function and the transfer function of the second hidden layer use “BFGS Quasi-Newton” and “sigmoid,” respectively. The parameter Net is the design result of the classifier.

The function “train ( )” is exploited to train the BPNN classifier. The input parameters *p_train* and *t_train* are the features and labels of the training set, respectively. The output parameter *Net_BP* is the predicted value of the BPNN after training.

The function “sim ( )” is engaged to test the BPNN classifier. The feature of the test set *p_test* is compared with the predicted value until the training parameters are satisfied, and the classification result *Error_sim_BP* is obtained.

##### 2.2.5.2 ELM classifier

The ELM classifier has the same structure as the BPNN classifier, and they both belong to the feed-forward neural network, while the hidden layer of ELM classifier is one. The weights and thresholds of the BPNN classifier are constantly changing, while the ELM classifier generate the unchanged weights and thresholds initially, which will save a lot of time compared to the training of BPNN classifier (Wang et al., 2021).

The key points in the building and training ELM classifier are the calculation of the connection weights (*IW*) between the hidden layer and the input layer, the thresholds (*B*) of the hidden layer neurons and the connection weights (*LW*) between the hidden layer and the output layer. Here, the number of nodes in the input layer, hidden layer, and output layer are 12, 300, and 5, respectively. In addition, *IW* and *B* are randomly generated by function “rand ( )” in MATLAB 2020b, where *IW* = rand (300,15) * 2–1, *B* = rand (300,1), and *LW* is calculated with the help of the function “pinv ( ).” The predicted value *Y* is computed by performing the classification using the sinusoidal transfer function based on parameters *IW*, *B*, and *LW*, and *Y* is equal to the inverse matrix of the inverse matrix of the output in the hidden layer (*H*) by *LW*; then, the maximum value of all the features of *Y* is used as the label, marked as 1, the others are 0, and the new predicted value is output.

##### 2.2.5.3 DT classifier

The DT has a top-down structure, growing down from the root to the nodes in a certain order to make a decision, and getting results at the leaves (Charbuty and Abdulazeez, 2021). The two functions of the DT classifier are as follows:

*Ctree* = fitctree (*p_train*, *t_train*);
*T_sim_tree* = predict (*Ctree*, *p_test*).

The function “fitctee ( )” is exploited to build and test the DT classifier, and the output parameter *Ctree* is the trained decision tree. The function “predict” is utilized to test the trained decision tree. Here, the feature space of the training and testing sets for the input parameters *p_train*, *t_train*, and *p_test* is different from that of BPNN and the ELM classifiers, which should be the number of samples ×feature properties.

## 3 Results

### 3.1 The preprocessing results

**FIGURE 6:**
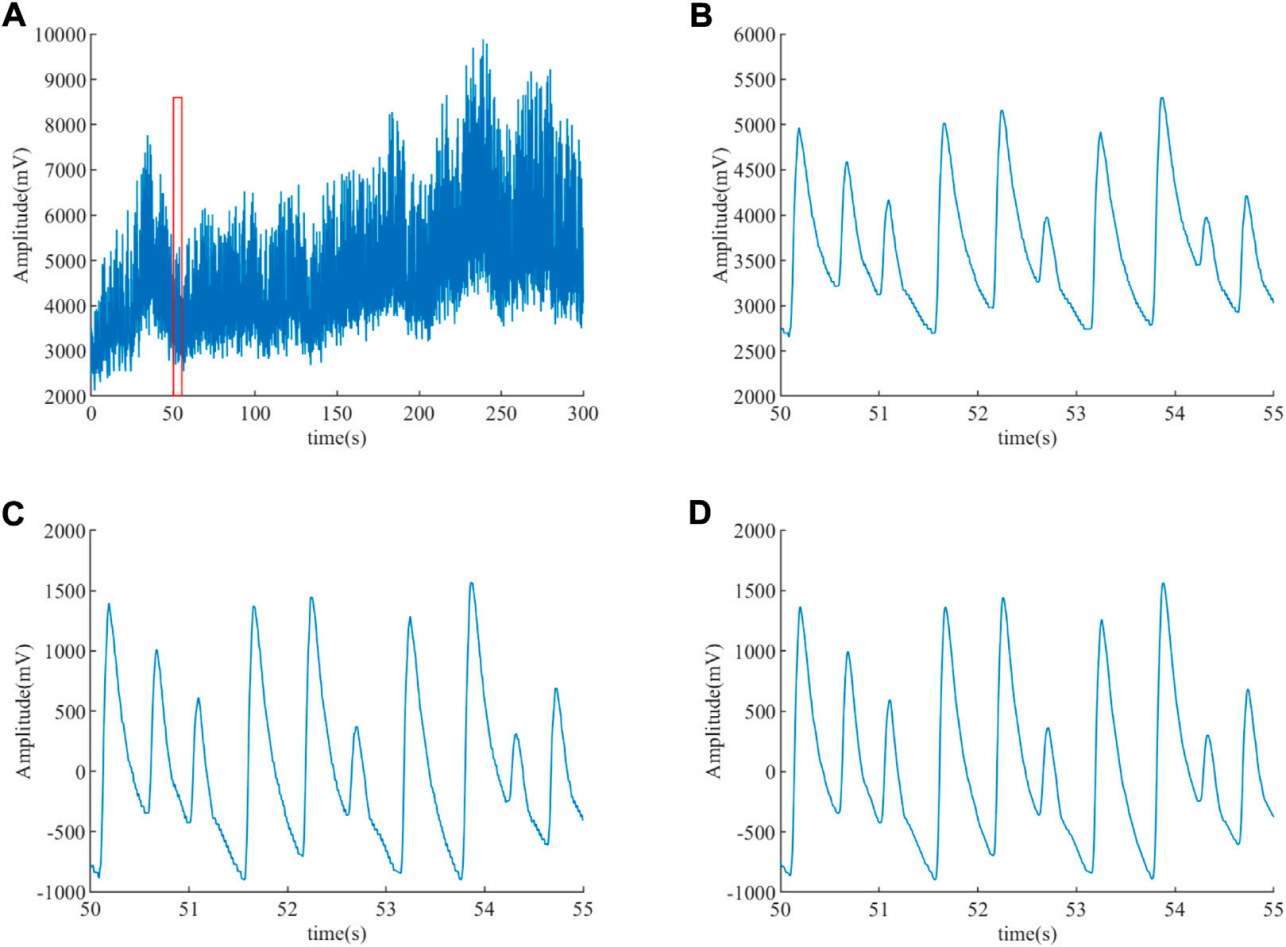
ABP signal before and after filtering for an ET patient. **(A)** ABP signal before filtering. **(B)** Enlargement of the red box **(A)**. **(C)** De-nosing the ABP signal by an integer coefficient notch filter. **(D)** De-nosing the ABP signal obtained **(C)** by an integer coefficient low-pass filter.

After de-noising the ABP signal using an integer coefficient notch filter and an integer coefficient low-pass filter, a clean ABP signal is obtained. Figure 6 displays the ABP before and after filtering for an ET patient. The AC interference and baseline drift are presented in Figure 6A, and the red box of Figure 6A is enlarged to Figure 6B in order to clearly observe these noises. It can be observed that the AC interference and the baseline drift have been wiped out in Figure 6C, the EMG interference has been eliminated in Figure 6D based on Figure 6C, and it can be clearly observed that the burr is eliminated in Figure 6C.

### 3.2 Pulse rate variability extraction results

**FIGURE 7:**
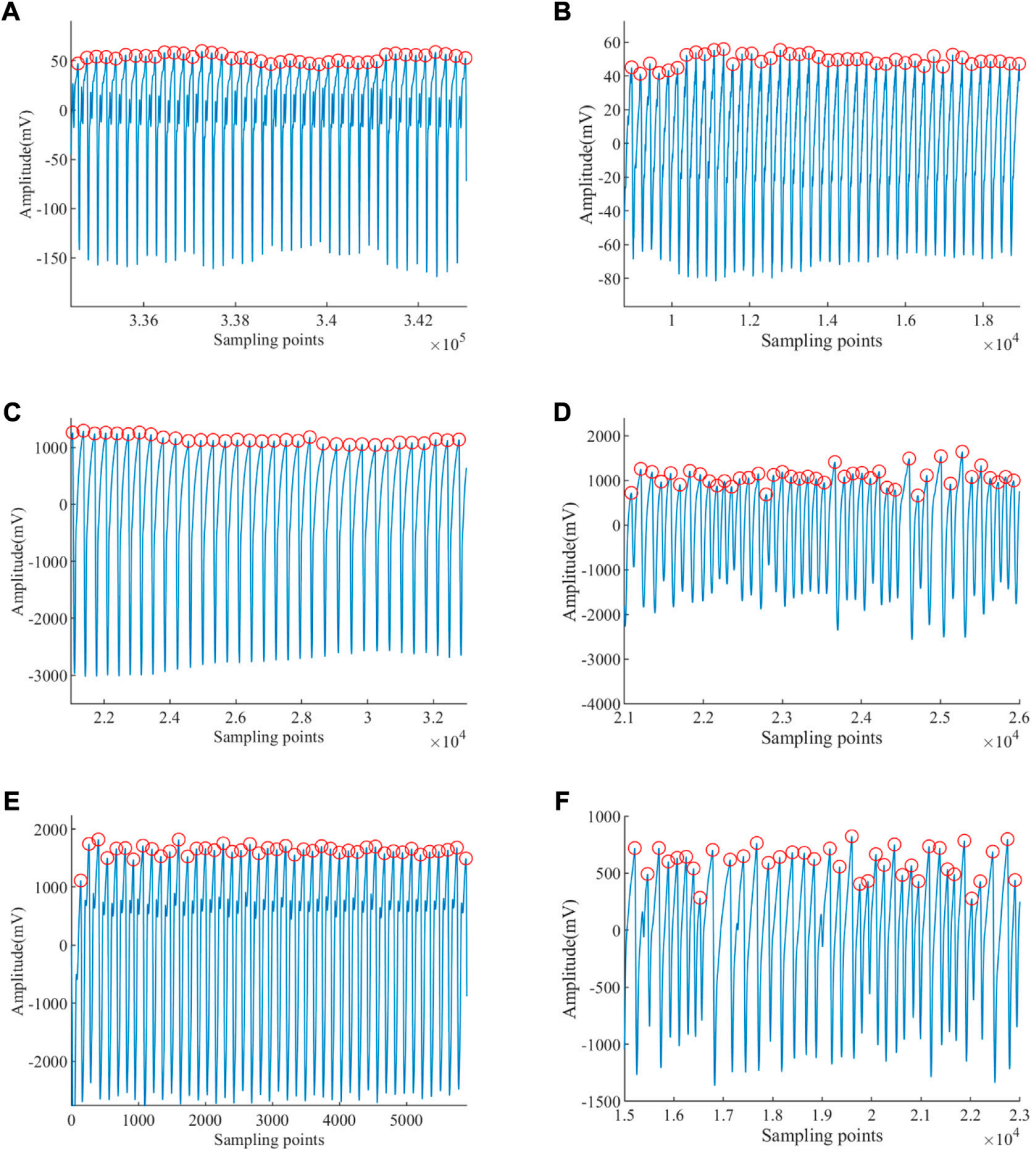
Peaks of ABP signal extracted. **(A)** Peak detection of a healthy young subject. **(B)** Peak detection of a healthy old subject. **(C)** Peak detection of an EB subject. **(D)** Peak detection of an ET subject. **(E)** Peak detection of a VT subject. **(F)** Peak detection of a VF subject.

The results of *Peaks* in subjects extracted from the ABP signal of different groups by the methods of SWIDFT and manual calibration are illustrated in Figure 7. Also, it can be observed that the method is highly accurate and robustly stable, which can be engaged effectively for PRV calculations.

**FIGURE 8:**
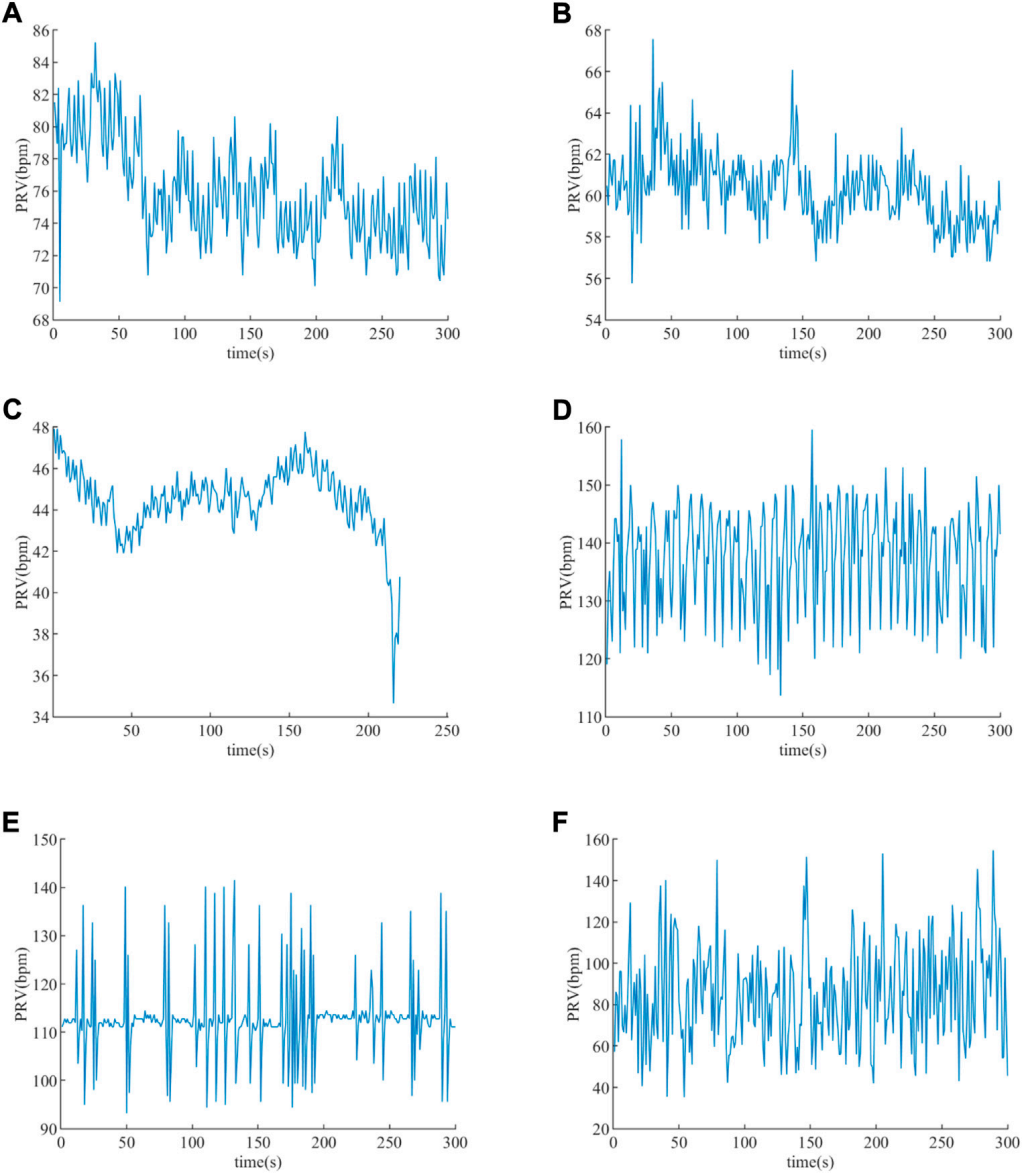
PRV extracted. **(A)** PRV of a healthy young subject. **(B)** PRV of a healthy old subject. **(C)** PRV of an EB subject. **(D)** PRV of an ET subject. **(E)** PRV of a VT subject. **(F)** PRV of a VF subject.

The PRV results of subjects extracted based on the *Peaks* detection are illustrated in Figure 8, from which it is obvious that the amplitudes of those PRV signals are different. The PRV signals’ average of healthy young, healthy old, EB, ET, VT, and VF are 75.902 beat per minute (bpm), 60.282 beat per minute (bpm), 44.462 bpm, 137.598 bpm, 112.760 bpm, and 84.714 bpm, respectively. The average heartbeat of EB is the lowest, while that of ET is the highest. The average heartbeat of VF is higher than that of the EB subjects and lower than that of the VT subjects.

### 3.3 Feature extraction results

A total of 19 features (defined in Section 2.2.3) were extracted from the PRV signal, and the statistical results of the features are presented as “mean ± standard deviation” in Table 2. In total, we extracted the 143853 PRV signal from the ABP signal, for which we calculated a feature vector of 19 × 139719. Among them, the feature vectors for healthy, EB, ET, VT, and VF subjects are 19 × 93516, 19 × 6475, 19 × 16124, 19 × 22083, and 19 × 1521, respectively.

**TABLE 2:** Results of feature extraction.

Feature	Healthy	EB	ET	VT	VF
Mean (bpm)	64.416 ± 9.927	68.415 ± 17.993	127.740 ± 25.192	97.849 ± 16.322	106.964 ± 40.108
Std (bpm)	4.225 ± 4.135	5.468 ± 5.469	19.756 ± 10.522	11.709 ± 8.557	25.690 ± 10.921
RMSD (bpm)	4.398 ± 5.280	6.022 ± 5.937	25.304 ± 12.951	15.603 ± 10.819	34.148 ± 14.017
NRMSD	0.070 ± 0.089	0.101 ± 0.106	0.201 ± 0.112	0.155 ± 0.101	0.327 ± 0.104
PNN40	0.282 ± 0.232	0.272 ± 0.284	0.509 ± 0.262	0.387 ± 0.274	0.809 ± 0.133
PNN70	0.116 ± 0.177	0.156 ± 0.222	0.364 ± 0.218	0.272 ± 0.230	0.712 ± 0.169
Mid (bpm)	63.982 ± 10.061	69.291 ± 17.707	128.012 ± 24.466	96.873 ± 16.795	106.532 ± 42.183
IQR	1.075 ± 0.082	1.105 ± 0.167	1.214 ± 0.181	1.116 ± 0.135	1.468 ± 0.351
LF_HF	0.690 ± 0.620	0.322 ± 0.396	0.326 ± 0.391	0.327 ± 0.391	0.358 ± 0.466
Sd1_Sd2	0.600 ± 0.285	1.008 ± 0.461	1.080 ± 0.502	1.075 ± 0.383	1.136 ± 0.524
Se	17,957.633 ± 48,104.424	84,636.350 ± 207,135.375	28,566.523 ± 33,315.262	48,468.728 ± 653,737.846	127,469.782 ± 153,701.838
TPR_PR	0.387 ± 0.102	0.298 ± 0.113	0.294 ± 0.107	0.277 ± 0.117	0.283 ± 0.099
ShE_PR	0.770 ± 0.263	0.733 ± 0.282	0.798 ± 0.232	0.677 ± 0.267	0.833 ± 0.219
SamE_PR	1.401 ± 0.541	0.924 ± 0.659	1.097 ± 0.777	0.888 ± 0.697	1.151 ± 0.752
C_ SamE_PR	−3.445 ± 0.565	−3.952 ± 0.721	−4.426 ± 0.777	−4.374 ± 0.727	−4.151 ± 0.754
PE_PR	5.145 ± 0.223	4.013 ± 0.399	4.158 ± 0.158	4.108 ± 0.207	4.174 ± 0.140
RMSD_APM	0.088 ± 0.062	0.245 ± 0.183	0.837 ± 0.397	0.610 ± 0.419	0.584 ± 0.224
SamE_APM	1.380 ± 0.498	1.106 ± 0.675	1.124 ± 0.816	1.071 ± 0.661	1.336 ± 0.697
TPR_APM	0.461 ± 0.090	0.381 ± 0.108	0.283 ± 0.096	0.272 ± 0.115	0.286 ± 0.126

### 3.4 Feature dimensionality reduction results

To reduce the complexity of the algorithm without affecting the accuracy as much as possible, the features extracted from the PRV signal need to be dimensionalized by the method of RF.

**FIGURE 9:**
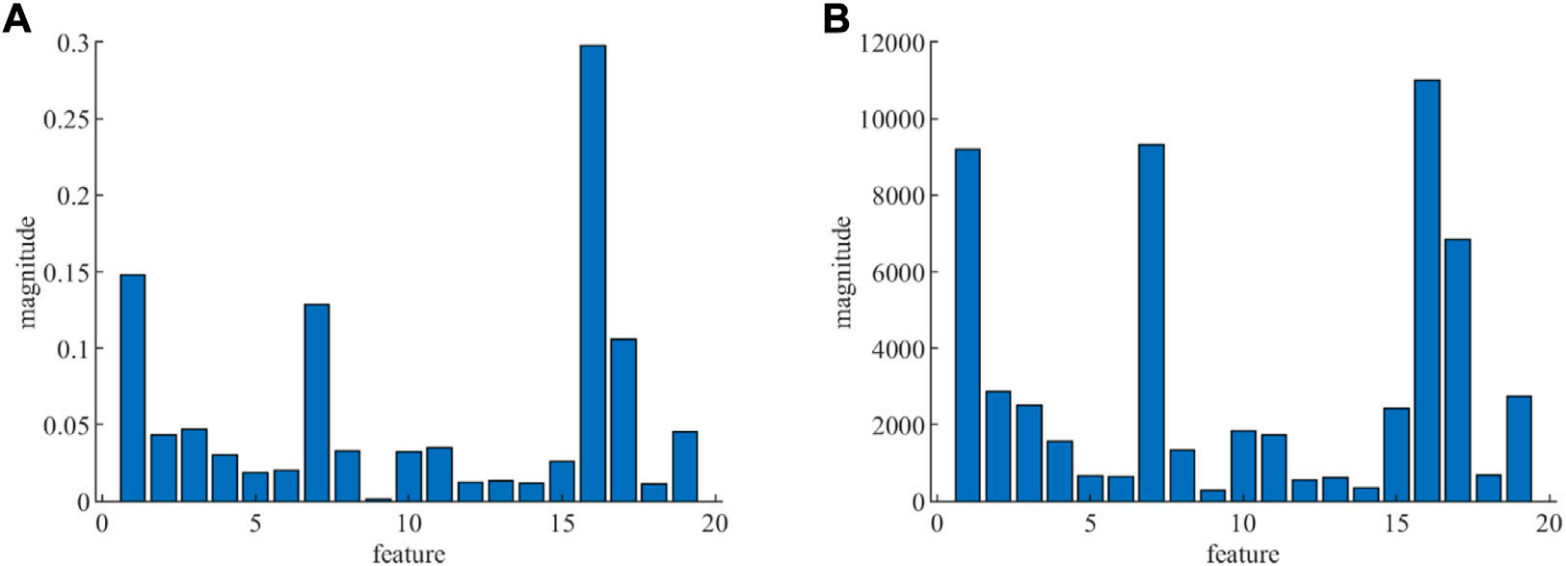
Magnitude of feature. **(A)** Mean decrease in accuracy. **(B)** Mean decrease in the Gini index.

The magnitude of the features calculated with RF is displayed in Figure 9, the mean decrease of accuracy is given in Figure 9A, and the mean decrease of Gini index is given in Figure 9B. For each feature, the trend of the Gini index and accuracy is essentially the same, which ensures the correctness of feature importance on both sides. The feature values of the accuracy and Gini index are illustrated in the third and fourth columns of Table 3. Table 3 displays the result of feature dimensionality reduction with RF, and the statistical results of healthy and four life-threatening arrhythmia patients are shown in column 2. According to Figure 8 and the feature values of accuracy and Gini index, 15 feature parameters are selected, which contains most of the information about the PRV signal. In addition, the results of feature selection (*h*) shows in the last column of Table 3, where *h* = 1 is the feature accepted and *h* = 0 is the feature rejected. Therefore, in this study, 15 features can be exploited to detect life-threatening arrhythmias, and the feature vector becomes 15 × 139719.

**TABLE 3:** Results of feature dimensionality reduction with RF.

Feature	Arrhythmia	Magnitude		
		Accuracy	Gini index	h
Mean (bpm)	77.656 ± 26.427	0.14803	9190.703	1
Std (bpm)	7.491 ± 8.342	0.04319	2875.464	1
RMSD (bpm)	8.981 ± 10.923	0.04695	2509.154	1
NRMSD	0.103 ± 0.109	0.03050	1566.921	1
PNN40	0.330 ± 0.261	0.01856	658.777	1
PNN70	0.178 ± 0.220	0.02003	650.410	1
Mid_PR	77.279 ± 26.823	0.12823	9310.805	1
IQR	1.103 ± 0.131	0.03286	1326.019	1
LF_HF	0.570 ± 0.581	0.00151	296.167	0
Sd1_Sd2	0.755 ± 0.411	0.03250	1834.461	1
Se	28286.557 ± 268061.587	0.03482	1744.127	1
TPR_PR	0.353 ± 0.116	0.01240	557.654	0
ShE_PR	0.758 ± 0.263	0.01369	623.074	1
SamE_PR	1.260 ± 0.642	0.01170	350.191	0
CSampEn	−3.736 ± 0.760	0.02585	2423.171	1
PE_PR	4.804 ± 0.535	0.29766	10999.265	1
RMSD_APM	0.269 ± 0.360	0.10571	6848.117	1
SamE_APM	1.288 ± 0.596	0.01161	676.207	0
TPR_APM	0.405 ± 0.127	0.04555	2737.818	1

### 3.5 Classification results

In this study, kappa coefficients (Islam et al., 2018) and accuracy (Sabut et al., 2021) were exploited to calculate the average performance of supervised learning to recognize the life-threatening arrhythmia results. The accuracy is calculated as follows

$$accuracy=\frac{TN+TP}{TN+FP+TP+FN}\times 100\%,$$ (29)

where the parameters “TN,” “FP,” “TP,” and “FN” are true negative, false positive, true positive, and false negative of the classification result, respectively.

The kappa coefficient (*kappa*) is calculated as follows

$$kappa=\frac{p_{1}-p_{2}}{1-p_{2}},$$ (30)

$$p_{1}=\frac{\sum_{t=1}^{r}Q_{tt}}{M},$$ (31)

$$p_{2}=\frac{\sum_{t=1}^{r}\left(Q_{t+}\times Q_{+t}\right)}{M^{2}}.$$ (32)

Moreover, the *kappa*(*i*) is utilized to evaluate the average performance of the classification results for the healthy and the four life-threatening arrhythmia subjects, and the *i* is the label of five types of subjects.

$$kappa\left(i\right)=\frac{P_{tt}-P_{t+}P_{+t}}{P_{+t}-P_{t+}P_{+t}},$$ (33)

$$P_{tt}=\frac{Q_{tt}}{M},$$ (34)

$$P_{t+}=\frac{Q_{t+}}{M},$$ (35)

$$P_{+t}=\frac{Q_{+t}}{M}.$$ (36)

where “*p*_*1*_” is the rate of correct classification, “*p*_*2*_” is the rate of incorrect classification, “*Q*_*tt*_” is the sum of elements on the diagonal of the column matrix, “*r*” is the number of features, “M” is the number of classes, and “*Q*_*t+*_” and “*Q*_*+t*_” are the sum of elements on *t*th row and column, respectively. Here, “*r*” and “*M*” are equal to 15 and 5, respectively. The kappa∈[-1,1], and the closer the value to 1, the better the classification result.

In Table 3, 15 features were selected by RF to form the feature vector. Therefore, the size of the feature vector becomes 15 × 139,719. We randomly selected 80 and 20 percent of the features as the training set and the test set, respectively. The features of the training and test sets were randomly changed 100 times to minimize the influence of input data differences, and the procedure was run 100 times in order to verify the classification accuracy of BPNN, ELM, and DT. The results of classification performance are displayed as “mean ± standard deviation” in Table 3, and 1, 2, 3, 4, and 5 are the labels for healthy, EB, ET, VT, and VF subjects, respectively.

As demonstrated in Table 4, the average performance of the classifier was verified with accuracy and kappa coefficient, whose result of BPNN is 94.85 ± 1.33% and 89.95 ± 2.62%, that of ELM is 95.05 ± 0.14% and 90.28 ± 0.28%, and that of DT is 98.76 ± 0.08% and 97.59 ± 0.15%. Therefore, the DT classifier has the best average performance in identifying those four life-threatening arrhythmias. In addition, the time consumption of BPNN is 100.58 ± 26.49 s, that of ELM is 8.63 ± 0.22 s, and that of DT is 1.12 ± 0.09 s. In brief, the performance of the DT classifier is optimal in the detection of the four arrhythmias. For identifying these life-threatening arrhythmias with the DT classifier, healthy subjects have the highest average performance with *kappa* (1) of 99.94 ± 0.05%, and VF patients have the lowest average performance with *kappa* (5) of 77.87 ± 2.39%. In addition, the average performance to detect EB, ET, and VF are all over 95.00%. With regard to time consumption, the DT and ELM classifiers take significantly less time than the BPNN classifier, which is because the BPNN classifier needs to constantly adjust the weights and thresholds.

**TABLE 4:** Classification results.

Classifier	BPNN	ELM	DT
Accuracy (%)	94.85 ± 1.33	95.05 ± 0.14	98.76 ± 0.08
Kappa (%)	89.95 ± 2.62	90.28 ± 0.28	97.59 ± 0.15
Kappa (1) (%)	99.60 ± 0.22	98.43 ± 0.22	99.94 ± 0.05
Kappa (2) (%)	93.02 ± 2.70	88.39 ± 1.18	98.70 ± 0.37
Kappa (3) (%)	80.43 ± 7.42	92.70 ± 0.69	96.87 ± 0.49
Kappa (4) (%)	82.22 ± 3.26	78.07 ± 0.81	95.46 ± 0.40
Kappa (5) (%)	72.51 ± 4.21	75.08 ± 2.45	77.87 ± 2.39
Time(s)	100.58 ± 26.49	8.63 ± 0.22	1.12 ± 0.09

## 4 Discussion

In this study, we propose a method to recognize four life-threatening arrhythmias based on the PRV signal calculated from the ABP signal of 2015 “PhysioNet/CinC” and “Fantasia” databases. A total of 19 features were extracted, and 15 of them were selected after feature dimensionality reduction to train and test the classifier. It can be illustrated that the DT classifier has the best average performance with *accuracy* and *kappa* of 97.59 ± 0.15% and 99.94 ± 0.05% in Table 4, respectively.

**FIGURE 10:**
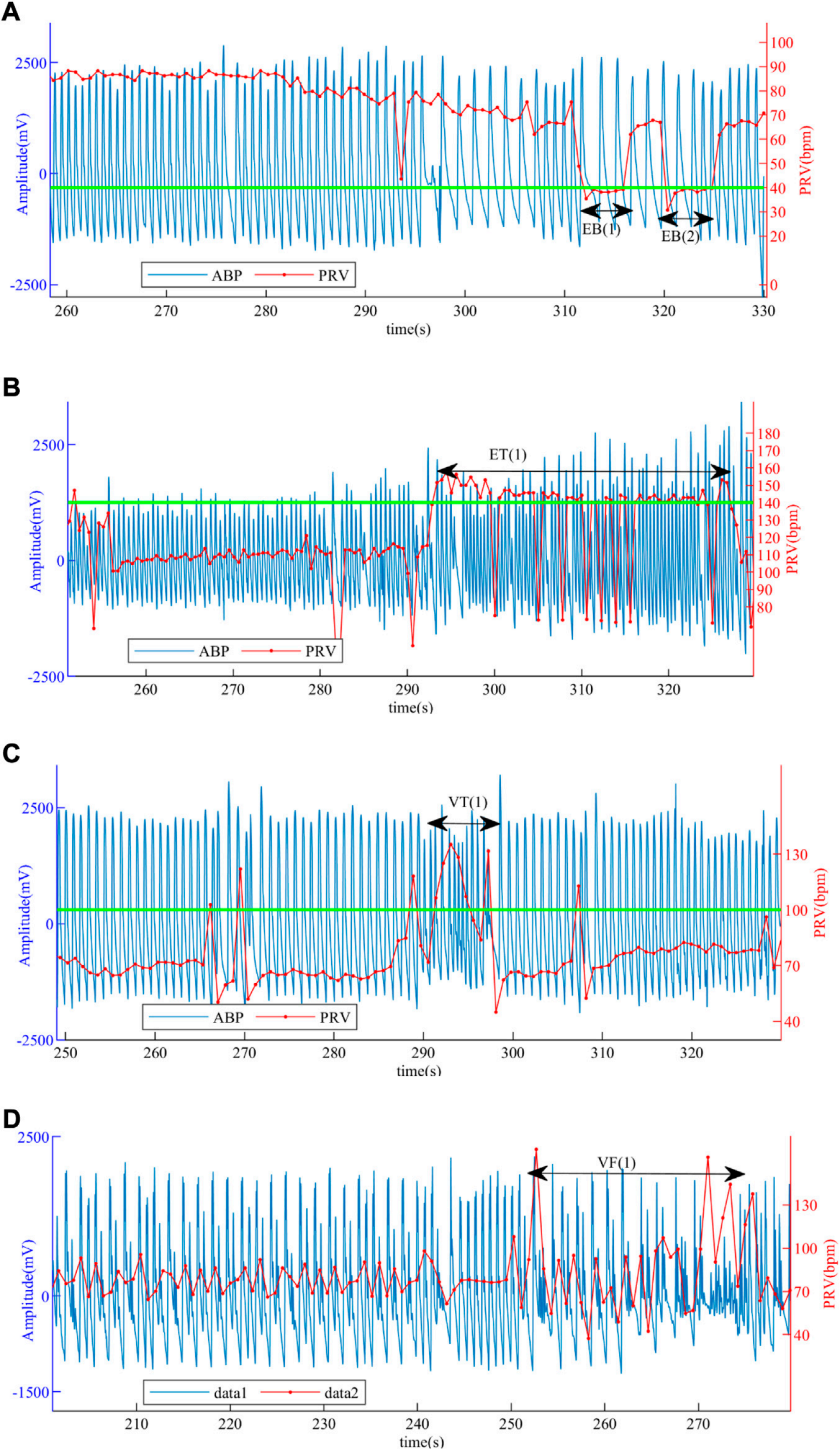
ABP and PRV signals of patients. **(A)** ABP and PRV signals of a patient with EB. **(B)** ABP and PRV signals of a patient with ET. **(C)** ABP and PRV signals of a patient with VT. **(D)** ABP and PRV signals of a patient with VF.

Figure 10 presents the ABP and PRV signals in different types of patients, where the green line is the standard of whether the disease is present or not, and the EB (1), EB (2), ET (1), VT (1), and VF (1) are the signals of those four life-threatening burst periods. In general, the sudden segment signals EB (1), EB (2), ET (1), VT (1), and VF (1) in patients are used for the recognition of life-threatening arrhythmias. However, since the “transient” of life-threatening arrhythmias can paralyze the patient and the signal can change rapidly and return to normal values, it is more important to confirm for the patient before the burst, which can alert the patients and send them to the hospital in time. The method we used detects not only the burst segment signal but also the normal segment signal before and after the burst, that is, the complete PRV signal in Figure 10, which is effective in identifying episodes of life-threatening arrhythmias.

So far, some researchers have studied the recognition of life-threatening arrhythmias. For example, Lee, K. et al. utilized feature parameters *RMSD* and *ShE* to identify AF (Lee et al., 2017), Eerikäinen, L.M. et al. used feature parameters *PNN40*, *PNN70*, *ShE*, *RMSD*, *nRMSD*, *SampEn*, and *CSampEn* to detect AF (Eerikäinen et al., 2018). Although these methods detect other cardiovascular diseases rather than those four life-threatening arrhythmias described in this study, they can provide ideas for our study. Therefore, the recognition of life-threatening arrhythmias is performed by the method used in this work for the extracted features of these researchers.

The average performance results of training and testing the DT classifier with the features extracted by Lee, K. et al. and Eerikäinen, L.M. et al. are displayed in Table 5. For the features extracted by Lee, K. et al., the performance of the DT classifier gives an *accuracy* of 83.32 ± 0.22% and *kappa* of 65.88 ± 0.42%, and the best average performance is ET with the kappa of 68.81 ± 0.92%. For the features extracted by Eerikäinen, L.M. et al., the performance of the DT classifier gives an *accuracy* of 95.27 ± 0.16% and *kappa* of 90.72 ± 0.32%, and the best average performance is healthy with the kappa of 95.90 ± 0.37%. In addition, the average performance of identifying those four life-threatening arrhythmias using the features utilized in Eerikäinen, L.M. et al. is better than that of Lee, K. et al., and the difference between them for *accuracy* and *kappa* is 11.95% and 24.84%, respectively, which is due to the fact that more features are engaged by Eerikäinen, L.M. et al. However, the accuracy and *kappa* values of Eerikäinen et al. are 3.49% and 6.87% lower than those of the method we used, which proves that the more features there are, the more comprehensive the information contained, and the higher the classification performance. However, it is not better to use more features if these features are correlated; it will cause a dimensional disaster which will affect the training of the model, reduce the average performance of the classification, and be more time consuming.

**TABLE 5:** Classification results of different features.

Feature	Lee, K. et al	Eerikäinen, L.M. et al
	*RMSD* and *ShE*	*PNN40*, *PNN70*, *ShE*, *RMSD*, *nRMSD*, *SampEn*, and *CSampEn*
*Accuracy* (%)	83.32 ± 0.22	95.27 ± 0.16
*Kappa* (%)	65.88 ± 0.42	90.72 ± 0.32
*Kappa* (1) (%)	67.94 ± 0.61	95.90 ± 0.37
*Kappa* (2) (%)	60.65 ± 1.83	87.25 ± 1.22
*Kappa* (3) (%)	68.81 ± 0.92	89.33 ± 0.63
*Kappa* (4) (%)	61.86 ± 0.99	86.00 ± 0.69
*Kappa* (5) (%)	49.39 ± 4.55	68.24 ± 2.76
Time(s)	0.53 ± 0.0	1.27 ± 0.12

## 5 Conclusion

In this study, a method is presented to identify four types of life-threatening arrhythmia identification based on the PRV signal. First, the noise of ABP signals is eliminated during preprocessing to de-noise the EMG interference, AC interference, and baseline drift. Then, PRV signals are extracted, and 15 features are obtained and downscaled from the PRV signal to form a feature vector. Finally, the BPNN, ELM, and DT classifiers are trained and tested based on the feature vector. The results show that DT has the best average performance with an accuracy of over 98.50% and a *kappa* of over 97.50%, which is better than some previous studies. Therefore, the method we used can effectively detect EB, ET, VT, and VF and has a potential for monitoring at home. In subsequent studies, the detection of motion artifacts will be added to the preprocessing part to improve the signal availability, and some algorithms such as feature extraction will be optimized. In the future, the DT model based on PRV signals is expected to be used for the recognition of other life-threatening arrhythmias.

## Data Availability

The datasets for this study can be found at the https://www.physionet.org/content/fantasia/1.0.0/ and https://www.physionet.org/content/challenge-2015/1.0.0/.
