# Corrigendum on: A life-threatening arrhythmia detection method based on pulse rate variability analysis and decision tree

**DOI:** 10.3389/fphys.2022.1102527

**Published:** 2022-11-29

**Authors:** Lijuan Chou, Jicheng Liu, Shengrong Gong, Yongxin Chou

**Affiliations:** ^1^ School of Electrical and Automatic Engineering, Changshu Institute of Technology, Suzhou, China; ^2^ School of Computer and Information Technology, Northeast Petroleum University, Daqing, China; ^3^ School of Computer Science and Engineering, Changshu Institute of Technology, Suzhou, China

**Keywords:** pulse rate variability, arterial blood pressure, cardiovascular diseases, life-threatening arrhythmias, decision tree, intelligent recognition

In the published article, there was an error in **Affiliation(s) [1]**. Instead of “[Country School of Electrical and Automatic Engineering, Changshu Institute of Technology, Suzhou, China],” it should be “[School of Electrical and Automatic Engineering, Changshu Institute of Technology, Suzhou, China].”

The authors apologize for this error and state that this does not change the scientific conclusions of the article in any way. The original article has been updated.

