# Supplementary material for: A life-threatening arrhythmia detection method based on pulse rate variability analysis and decision tree
Source: Front Physiol. 2022 Oct 14;13:1008111. doi: 10.3389/fphys.2022.1008111 (PMC9614148; doi:10.3389/fphys.2022.1008111)
Supplement: Supplementary file 1 [file DataSheet1.pdf]

# Supplementary Material

## 1 Supplementary Data

The datasets for this study can be found at the https://www.physionet.org/content/fantasia/1.0.0/ and https://www.physionet.org/content/challenge-2015/1.0.0/.

**Supplementary Table 1.** The datas of healthy subjects

| Old | | | Young | | |
|---|---|---|---|---|---|
| Name | Gender | Age | Name | Gender | Age |
| f2o01m | female | 73 | f2o01m | female | 23 |
| f2o02m | female | 75 | f2o02m | male | 23 |
| f2o03m | female | 85 | f2o03m | female | 28 |
| f2o04m | female | 70 | f2o04m | female | 27 |
| f2o05m | male | 83 | f2o05m | female | 25 |
| f2o06m | male | 70 | f2o06m | male | 26 |
| f2o07m | male | 77 | f2o07m | male | 31 |
| f2o08m | male | 71 | f2o08m | male | 21 |
| f2o09m | male | 77 | f2o09m | female | 21 |
| f2o010m | female | 73 | f2o010m | male | 21 |

**Supplementary Table 2.** The datas of life-threatening arrhythmias subjects

| EB | | ET | | VT | | | VF |
|---|---|---|---|---|---|---|---|
| b268s | b515l | t110s | t173l | v131l | v133l | v648s | f544s |
| b455l | b516s | t174s | t208s | v197l | v221l | v724s | f563l |
| b456s | b517l | t175l | t209l | v253l | v254s | v729l | f450s |
| b494s | b560s | t213l | t214s | v255l | v290s | v765l | f543l |
| b495l | b561l | t279l | t251l | v309l | v318s | v788s | f545l |
| b562s | b578s | t252s | t276s | v328s | v329l | v797l | f697l |
| b659l | b664s | t277l | t284s | v334s | v522s | v701l | |
| b708s | b722s | t305l | t333l | v523l | v541l | v726s | |
| b757l | | t335l | t406s | v542s | v559l | v758s | |
| | | t412s | t413l | v564s | v571l | v772s | |
| | | t417l | t418s | v573l | v579l | v793l | |
| | | t424s | t425l | v596s | v607l | v803l | |
| | | t594s | t677l | v625l | v626s | v788s | |
| | | t690s | t702s | v629l | v646s | v797l | |
| | | t707l | t719l | v793l | v842s | v813l | |
| | | t731l | t739l | v803l | v836s | v831l | |
| | | t760s | t777l | v815l | v844s | v837l | |
| | | t816s | | | | | |
